# Endangered Lymphocytes: The Effects of Alloxan and Streptozotocin on Immune Cells in Type 1 Induced Diabetes

**DOI:** 10.1155/2021/9940009

**Published:** 2021-10-19

**Authors:** Luiz A. D. Queiroz, Josiane B. Assis, João P. T. Guimarães, Emanuella S. A. Sousa, Anália C. Milhomem, Karen K. S. Sunahara, Anderson Sá-Nunes, Joilson O. Martins

**Affiliations:** ^1^Laboratory of Immunoendocrinology, School of Pharmaceutical Sciences, Department of Clinical and Toxicological Analyses, University of São Paulo, São Paulo, SP, Brazil; ^2^Laboratory of Experimental Immunology, Institute of Biomedical Sciences, Department of Immunology, University of São Paulo, São Paulo, SP, Brazil; ^3^Institute of Tropical Pathology and Public Health, Department of Microbiology, Immunology, Parasitology and Pathology, Federal University of Goiás, Goiânia, GO, Brazil; ^4^Experimental Physiopathology, Department of Sciences/Experimental Physiopathology, Medical School, University of São Paulo, São Paulo, SP, Brazil

## Abstract

Alloxan (ALX) and streptozotocin (STZ) are extensively used to induce type 1 diabetes (T1D) in animal models. This study is aimed at evaluating the differences in immune parameters caused by ALX and STZ. T1D was induced either with ALX or with STZ, and the animals were followed for up to 180 days. Both ALX and STZ induced a decrease in the total number of circulating leukocytes and lymphocytes, with an increase in granulocytes when compared to control mice (CT). STZ-treated mice also exhibited an increase in neutrophils and a reduction in the lymphocyte percentage in the bone marrow. In addition, while the STZ-treated group showed a decrease in total CD3^+^, CD4^−^CD8^+^, and CD4^+^CD8^+^ T lymphocytes in the thymus and CD19^+^ B lymphocytes in the pancreas and spleen, the ALX group showed an increase in CD4^−^CD8^+^ and CD19^+^ only in the thymus. Basal levels of splenic interleukin- (IL-) 1*β* and pancreatic IL-6 in the STZ group were decreased. Both diabetic groups showed atrophy of the thymic medulla and degeneration of pancreatic islets of Langerhans composed of inflammatory infiltration and hyperemia with vasodilation. ALX-treated mice showed a decrease in reticuloendothelial cells, enhanced lymphocyte/thymocyte cell death, and increased number of Hassall's corpuscles. Reduced *in vitro* activation of splenic lymphocytes was found in the STZ-treated group. Furthermore, mice immunized with ovalbumin (OVA) showed a more intense antigen-specific paw edema response in the STZ-treated group, while production of anti-OVA IgG1 antibodies was similar in both groups. Thereby, important changes in immune cell parameters *in vivo* and *in vitro* were found at an early stage of T1D in the STZ-treated group, whereas alterations in the ALX-treated group were mostly found in the chronic phase of T1D, including increased mortality rates. These findings suggest that the effects of ALX and STZ influenced, at different times, lymphoid organs and their cell populations.

## 1. Introduction

Diabetes mellitus is a chronic disorder characterized by persistent levels of hyperglycemia caused by an insufficient production of insulin by the *β* cells of the pancreas due to a destruction of these cells in type 1 diabetes (T1D) or by ineffective insulin action in type 2 diabetes (T2D) [1–3]. Rodents have been extensively used as diabetes experimental models [4]—chemically induced diabetes is mainly useful for studying T1D. Alloxan (ALX) and streptozotocin (STZ) bind to the glucose transporter- (GLUT-) 2 receptor, causing cell death by reactive oxygen species (ROS) generation (ALX) or inducing DNA damage (STZ) directly [5]. These diabetogenic agents, however, may have different toxicological effects depending on the dose and route of administration [6]. Although they have been used for quite a long time, the immunotoxicological effects of ALX and STZ are not fully clear yet.

One of the obnoxious effects of STZ is its toxicity to lymphocytes, diminishing T cell proliferation [7, 8], reducing the CD8^+^ T cell population in the blood and causing lymphopenia in the spleen and blood [7]. Gaulton et al. compared the toxicity of ALX and STZ in immune cells, both *in vivo* and *in vitro*, and found a greater impairment of lymphocyte function even when STZ was administered in doses lower than necessary for T1D onset. In contrast, a 10-fold increase in the ALX dose was not harmful to lymphoid cells [9]. Diab et al. conducted a similar study to evaluate the cytotoxicity of both agents, finding *in vitro* changes in blood cell populations, reduction of splenocytes, and immunosuppressive effects on graft transplantation in individuals treated with STZ [10].

A database search carried out by Muller et al. showed that in 131 articles on murine islet transplantation, 76.3% used the STZ diabetic model, while 3.8% used ALX [7]. STZ is the preferred model because it has a longer half-life, with prolonged hyperglycemia and lower mortality rates [10, 11]. Although less expensive, ALX is very unstable and may induce a reversible hyperglycemia that is undesirable and sometimes lethal [12].

Since the study of immune cell responses is of paramount significance/importance in T1D models, the comparison of different immune cell variables is vital to better understand the behavior of these cells and how they could be influenced by the choice of diabetogenic agents. To clarify these effects, this study is aimed at evaluating the differences in immune parameters caused by ALX and STZ, with special attention to T cell phenotype and function in lymphoid and nonlymphoid tissues.

## 2. Methods

### 2.1. Animal Model

Wild-type C57BL/6J male mice (12-14 weeks old, 25 ± 2 g at baseline) were housed at 22°C under a 12/12-hour light-dark cycle and given *ad libitum* access to food and water. This study was carried out in strict accordance with the principles and guidelines of the National Council for the Control of Animal Experimentation (CONCEA) and approved by the Ethics Committee on Animal Use (CEUA) at the Faculty of Pharmaceutical Sciences, University of São Paulo (FCF/USP), Brazil (protocol number: CEUA/FCF/338).

### 2.2. Induction of Diabetes

The animals were divided into three groups: (1) wild-type C57BL/6J control mice (CT), (2) ALX-treated mice, and (3) STZ-treated mice. Briefly, to induce T1D with ALX, the animals were fasted for 12 hours, followed by an intravenous (i.v.) injection of 60 mg/kg of alloxan monohydrate (Sigma-Aldrich, San Louis, MO, USA) dissolved in 100 *μ*L of sterile saline solution (0.9% NaCl) [13] using insulin syringes with 12.7 mm × 29G needle (BD Ultra-Fine, Franklin Lakes, New Jersey, USA); for STZ, the animals were fasted for 5 hours, followed by an intraperitoneal (i.p.) injection of 65 mg/kg of streptozotocin (ChemCruz®, Santa Cruz, CA, USA) dissolved in 300 *μ*L of 0.1 M citrate buffer, pH 4.5, for 5 consecutive days [3], using insulin syringes with 12.7 mm × 29G needle. After 15 days from the start of the induction protocol, the glycemia of the animals was measured using Accu-Chek Advantage II (Roche Diagnostics, São Paulo, SP, Brazil). Only the animals with glycemia above 300 mg/dL were considered diabetic for this study.

### 2.3. Physical Observation

In one set of experiments, daily observation throughout the study was carried out for mortality and general well-being in all groups. The consumption of water and food was monitored by weighing the average amount of food (g) and water (mL) consumed per mouse, for 5 days [14]. Following diabetes induction, glycemia and body weight were monitored at various time points (15^th^, 30^th^, 60^th^, 90^th^, and 180^th^ days) and compared to CT mice.

### 2.4. Insulin Tolerance Test (ITT)

Insulin tolerance tests were performed after 6 hours and on the 90^th^ and 180^th^ days after inducing T1D. Initial blood glucose levels were determined followed by intraperitoneal injection of human insulin (Humulin®, Fegersheim, France) (0.75 unit/kg) using insulin syringes with 12.7 mm × 29G needle. Blood glucose levels were measured via tail vein blood at 5, 10, 15, 20, 25, and 30 minutes after the injection [15–17].

### 2.5. Glucose Tolerance Test (GTT)

Glucose tolerance tests were performed after an overnight fast (12 hours) and on the 90^th^ and 180^th^ days after inducing T1D. Initial blood glucose levels were determined followed by intraperitoneal injection of glucose solution (Thermo Fisher Scientific, Rockford, IL, USA) (1 g/kg) using insulin syringes with 12.7 mm × 29G needle. Blood glucose levels were measured via tail vein blood at 15, 30, 60, 90, and 120 minutes after the injection [16, 18].

### 2.6. Hematological Parameters

Blood samples were collected via a facial plexus route on the 15^th^, 30^th^, 90^th^, and 180^th^ days after inducing T1D. Samples of EDTA-anticoagulated blood (1 : 10) were used to determine the following hematological parameters [19]: red blood cells (RBC), hemoglobin (HGB), hematocrit (HCT), mean corpuscular volume (MCV), mean corpuscular hemoglobin (MCH), mean corpuscular hemoglobin concentration (MCHC), red cell distribution width (RDW), platelets (PLT), leukocytes (WBC), lymphocytes (Lymph), monocytes (Mon), granulocytes (Gran), and the percentage of lymphocytes (Lymph%), monocytes (Mon%) and granulocytes (Gran%). All analyses were performed using an automated hematology counter (BC-2800Vet Mindray, Shenzhen, GD, China).

Bone marrow was collected from femurs on the 15^th^ day after inducing T1D, and cell suspensions were centrifuged in glass slides at 400 × *g*, 4°C, for 5 minutes, using a Cytospin centrifuge (Thermo Fisher Scientific), and allowed to dry for 20 minutes at room temperature [20]. The slides were stained using fast panoptic (LB Laborclin, Pinhais, PR, Brazil). A total of 100 cells were counted based on morphological criteria, classified as either mononuclear or polymorphonuclear [21, 22], using a conventional optical microscope (CX31RBSFA, Tokyo, Japan).

### 2.7. Histopathological Analysis

Histopathological analyses were performed in fragments of thymus, spleen, and pancreas tissue collected on the 15^th^ day after inducing T1D. Samples were fixed in a solution of 10% paraformaldehyde, dehydrated with alcohol, diaphanized in xylol, and embedded in paraffin. Blocks were cut into 3 *μ*m width slices, and fragments were captured with glass slides and stained with hematoxylin and eosin (H&E) [23].

General toxicity processes were described and classified according to structural changes by a semiquantitative analysis, as follows: absent, when there was no compromise of the tissue, score = 0; discrete, with up to 25% of area commitment, score = 1; moderate, from 26% to 50% of area commitment, score = 2; and accentuated, with more than 50% of area commitment, score = 3 [23–25].

### 2.8. Spleen and Pancreas Homogenates

Tissue samples of spleen and pancreas were separately collected from the mice on the 15^th^ day after inducing T1D and homogenized in radioimmunoprecipitation assay (RIPA) buffer (50 mM Tris, pH 8.0, 150 mM NaCl, 1% Triton X-100, and 0.1% SDS) containing a protease inhibitor (Sigma-Aldrich), with a tissue homogenizer (Polytron PT 1600E, Cincinnati, OH, USA). Supernatants were separated from the cellular debris by centrifugation at 239 × *g* for 5 minutes, collected and stored at −80°C.

Protein concentration in the homogenates was determined using Pierce™ BCA Protein Assay Kit (Thermo Fisher Scientific), according to the manufacturer's instructions.

### 2.9. Cytokine Determination

ELISA was used to determine the concentration of the following cytokines: interleukin- (IL-) 1*β*, IL-4, IL-12p70, tumor necrosis factor- (TNF-) *α*, and interferon- (IFN-) *γ* (Duo-set ELISA, R&D Systems Inc., Minneapolis, MN, USA); IL-2, IL-6, and IL-10 (BD OptEIA™ ELISA Set, BD Biosciences, San Diego, CA, USA); and IL-17A (ELISA MAX Deluxe Set, Biolegend, San Diego, CA, USA) in pancreas and spleen homogenates on the 15^th^ day after inducing T1D. The assays were performed according to the manufacturers' instructions.

### 2.10. Flow Cytometry

Following euthanasia, pancreas, spleen, and thymus were removed on the 15^th^ day after inducing T1D and macerated through a 40 *μ*m cell strainer. Spleen cell suspension was lysed using ACK Lysing Buffer (Gibco, Grand Island, NY, USA), and cell suspensions containing 1.5 × 10^6^ cells/mL were prepared in PBS containing 1% fetal bovine serum (FBS) and stained at 4°C for 30 minutes with fluorochrome-conjugated monoclonal antibodies against the following molecules (cell clone): CD3-PE (17A2), CD19-PE-Cy7 (6D5), CD25-PB (PC61), and CD11b-APC-Cy7 (M10/70) (Biolegend) and CD4-APC (RM4-5) and CD8-PE-Cy5 (53-6.7) (BD Biosciences). The cells were acquired in a FACSCanto II cytometer (BD Biosciences), and analysis was performed using the FlowJo software, version 10.0.7 (Tree Star, Ashland, OR, USA).

### 2.11. Immunization of Mice and Induction of Antigen-Specific Paw Edema

In another set of experiments, mice received three subcutaneous (s.c.) immunizations, with a 15-day interval between each one, consisting of 20 *μ*g/mice of OVA (Sigma-Aldrich) emulsified in 100 *μ*L of squalene adjuvant (Sigma-Aldrich). Doses were administered with a 15-day interval from one another [26]. Thirty days after the last immunization, the mice were submitted to the protocol of diabetes induction with either ALX or STZ.

The diabetic animals, together with the immunized CT and naïve mice, were challenged with s.c. injection of OVA (10 *μ*g in 30 *μ*L of saline) on the right paw. As a control, the mice also received a s.c. injection of 30 *μ*L saline on the left paw. Footpad thickness was measured immediately before and 6 hours after the inoculation using a caliper (Mitutoyo, Kawasaki, OL, Japan). The results are expressed as the mean difference between the measurements, as described [27].

### 2.12. Determination of OVA-Specific Anti-IgG1 and Anti-IgG2a Antibodies

After the immunization and antigenic challenge described above, blood samples were collected to determine OVA-specific anti-IgG1 and anti-IgG2a antibodies. The plates were coated overnight at 4°C with OVA (20 *μ*g/mL) in sodium carbonate buffer (pH 9.5), washed with PBS/0.05% Tween-20, and blocked with assay diluent PBS/10% FCS for 1 hour. Following washing, serum samples were added to the plates and incubated at room temperature (RT) for 2 hours. After new washing, anti-IgG1 or anti-IgG2a (BD Biosciences) conjugated with peroxidase was added and incubated for 1-hour RT. After washing, 3,3′,5,5′-Tetramethylbenzidine (TMB) Substrate Reagent Set (BD Biosciences) was added and the plates were left for 30 minutes RT in the dark [26]. Colorimetric reaction was stopped by adding 2 NH2SO4. Absorbance was acquired at 450 nM in the microplate reader SpectraMax 190. The data are shown in optical density (O.D) units.

### 2.13. Spleen Cell Proliferation

Spleen cell proliferation was evaluated at different stages of the study and by different methodologies. In all cases, spleens were removed, individually macerated through a 40 *μ*m cell strainer, and the cell suspensions were lysed using ACK Lysing Buffer (Gibco) before the respective assay.

On the 15^th^ day after inducing T1D in naïve mice, spleen cell suspensions containing 10^7^ cells/mL were stained with carboxyfluorescein succinimidyl ester (CFSE)-FITC (eBioscience, San Diego, CA, USA) according to the manufacturer's protocols. The cells were plated with complete medium (RPMI 1640 supplemented with 10% FBS, 1% penicillin/streptomycin, 2 mM L-glutamine, and 25 mM HEPES, all from Gibco) and stimulated with Concanavalin A (Con A—0.5 and 1 *μ*g/mL, Sigma-Aldrich) for 72 hours at 37°C and 5% CO_2_ [26]. After that, the cells were stained at 4°C for 30 minutes with anti-mouse CD4-APC and CD8-PE-cy5 (BD Biosciences), acquired in a FACSCanto II cytometer and analyzed as described above.

In mice immunized with OVA, spleen cell suspensions containing 10^6^ cells/mL were prepared in complete medium and stimulated with Con A (0.5 and 1 *μ*g/mL, Sigma-Aldrich) for polyclonal activation or with OVA (1 and 10 *μ*g/mL, Sigma-Aldrich) for antigen-specific proliferation. Proliferation was evaluated by a colorimetric assay using resazurin (Sigma-Aldrich), as previously described [28]. The results are expressed as the difference between the absorbance reading at 570 and 600 nm by a spectrophotometer (SpectraMax 190, San Jose, CA, USA) [29, 30].

### 2.14. Statistical Analysis

Statistical analyses were performed using the GraphPad 6 software (San Diego, CA, USA), and the data are presented as mean ± standard error of the mean (SEM) using analysis of variance (ANOVA), two-way for GTT and ITT and one-way for the other evaluations, followed by Bonferroni's multiple comparison test when appropriate. The significance level was set at *p* ≤ 0.05.

## 3. Results

### 3.1. ALX and STZ Increased Blood Glucose Levels, Induced Bodyweight Loss, and Changed Blood Cell Counts

**Figure 1:**
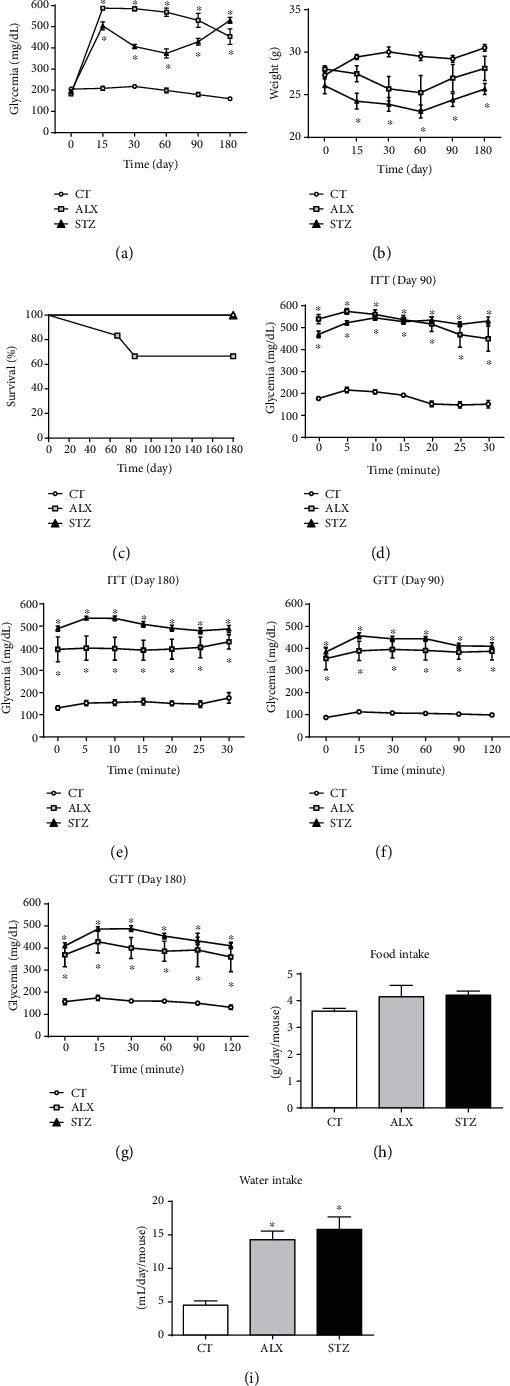
Long-term diabetogenic effects of ALX and STZ on mice. T1D was induced as described in Methods, and parameters were evaluated at different time points. (a) Blood glucose levels and (b) body weight were evaluated on the 15^th^, 30^th^, 60^th^, 90^th^, and 180^th^ days following administration of the drugs. (c) Survival rate was monitored for 180 days (CT and STZ groups represented as overlapping lines). (d, e) Insulin tolerance test and (f, g) glucose tolerance test were evaluated on the 90^th^ and 180^th^ days. (h) Food and (i) water intake was evaluated for five days. Data are presented as mean ± SEM, ^∗^*p* ≤ 0.05 (5-6 animals per group).

The effects of ALX and STZ treatment for T1D induction in mice were identified by measuring body weight and glycemia after a 15-day protocol. Both ALX- and STZ-treated animals exhibited glycemia above 300 mg/dL until the 180^th^ day, with the ALX-induced animals maintaining higher levels of hyperglycemia until the 90^th^ day (Figure 1(a)). The group treated with STZ showed a greater and more pronounced weight loss than the ALX group during the whole period evaluated (Figure 1(b)). Both diabetic groups showed an increase in water intake (Figures 1(i)), but not food intake (Figures 1(h)) compared to the CT group. After 180 days from T1D induction, there was no signal of insulin or glucose tolerance in the ALX and STZ groups (Figures 1(d)–1(g)). However, some animals from the ALX group started to die after 60 days (Figure 1(c)) and presented tumor nodules throughout the body (data not shown), as it has already been reported by other authors [31–33].

**Figure 2:**
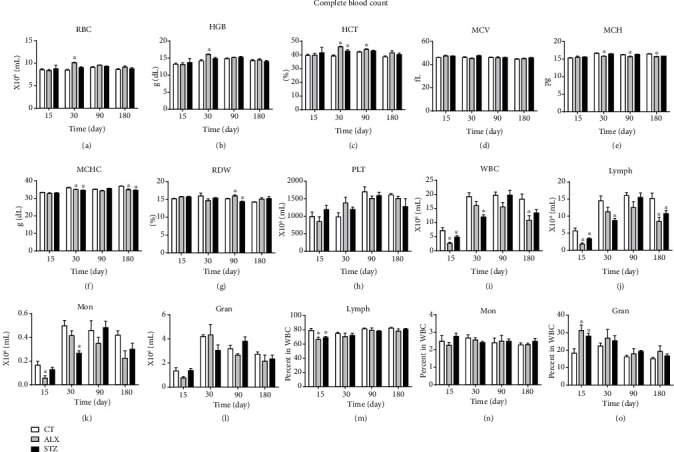
STZ effects on hematimetric parameters of immunological cells in the early phase of T1D are restored in the chronic phase of the disease. T1D was induced as described in Methods, and blood samples were collected in EDTA on the 15^th^, 30^th^, 90^th^, and 180^th^ days, to evaluate the following parameters: (a) red blood cells; (b) hemoglobin; (c) hematocrit; (d) mean corpuscular volume; (e) mean corpuscular hemoglobin; (f) mean corpuscular hemoglobin concentration; (g) red cell distribution width; (h) platelets; (i) leukocytes; (j) lymphocytes; (k) monocytes; (l) granulocytes; (m) percentage of lymphocytes; (n) percentage of monocytes; (o) percentage of granulocytes. Data are presented as mean ± SEM, ^∗^*p* < 0.05 (11-13 animals per group).

In order to explore the impacts of ALX and STZ on the population of blood cells in the early and chronic phases of T1D, we analyzed the whole blood count at different time points—15^th^, 30^th^, 90^th^, and 180^th^ days—and identified that both the ALX- and STZ-treated groups revealed a decrease in leukocytes (Figure 2(i)) and lymphocytes (Figure 2(j)) on the 15^th^ day. A lower percentage of lymphocytes (Figure 2(m)) and an increased percentage of granulocytes (Figure 2(o)) were found on the first 15 days in both the T1D-induced groups; despite fluctuating during the study, the number of lymphocytes remained lower in the last measurement (180^th^ day) when compared to the CT group (Figure 2(j)). The total leukocyte count varied over the study period. For monocytes, however, while ALX lowered their count by the 15^th^ day, this alteration only occurred after the 30^th^ day in the STZ-induced group (Figure 2(k)). ALX-induced animals showed consistently increased parameter values on the 30^th^ day for red blood cell count (Figure 2(a)), hemoglobin (Figure 2(b)), and hematocrit (Figure 2(c)), while their mean corpuscular hemoglobin concentration (Figure 2(f)) was lower in the same period. In addition, a differential count of nucleated cells in the bone marrow showed a decreased percentage of lymphocytes in the STZ-induced group in comparison with CT and ALX groups, suggesting its toxicity (Table 1). In parallel, the STZ-induced group presented an increased percentage of neutrophils in the bone marrow when compared to the other groups (Table 1).

### 3.2. STZ Impaired the Basal Production of IL-1*β* in the Pancreas and IL-6 in the Spleen

**Figure 3:**
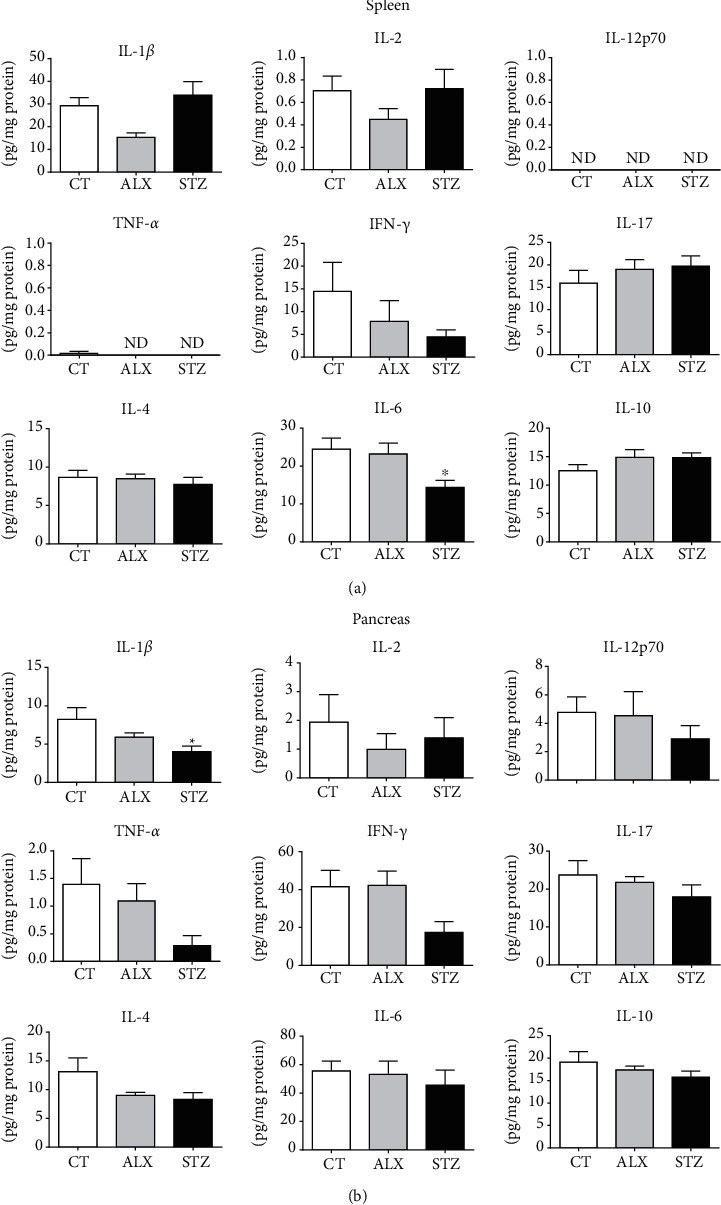
Short-term diabetogenic effects of ALX and STZ on the basal levels of cytokines in spleen and pancreas homogenates. T1D was induced as described in Methods, and (a) spleens and (b) pancreases were collected on the 15^th^ day. Tissue homogenates from the CT, ALX-, and STZ-treated groups were evaluated for the presence of the cytokines IL-1*β*, IL-2, IL-4, IL-6, IL-10, IL-12p70, IL-17, TNF-*α*, and IFN-*γ*. Data are presented as mean ± SEM, ^∗^*p* ≤ 0.05 (5-6 animals per group).

To assess whether ALX and STZ can influence the immunological steady-state profile of the animals, the basal levels of IL-1*β*, IL-2, IL-4, IL-6, IL-10, IL-12, IL-17, IFN-*γ*, and TNF-*α* in spleen (Figure 3(a)) and pancreas (Figure 3(b)) homogenates were measured. A significant reduction of IL-6 in the spleen and IL-1*β* in the pancreas in the STZ group was found, while all other cytokines were not altered. This could be a result of an imbalance of secretory cells due to the hyperglycemic environment rather than a toxicity of STZ.

### 3.3. Lymphocyte Populations in the Thymus, Spleen, and Pancreas Were Affected by STZ

**Figure 4:**
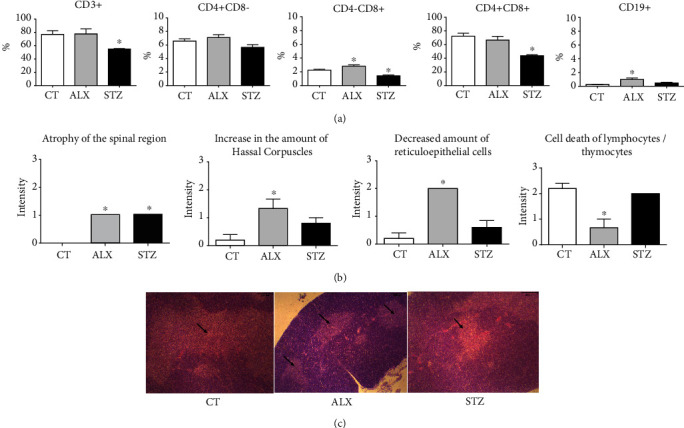
Short-term diabetogenic effects of ALX and STZ on the thymus. T1D was induced as described in Methods, and (a) thymuses from the CT, ALX, and STZ groups were collected on the 15^th^ day and characterized according to the percentage of CD3^+^, CD4^+^CD8^−^, CD4^−^CD8^+^, CD4^+^CD8^+^, T lymphocytes, and CD19^+^ B lymphocytes. (b) Evaluation of structural changes in the thymus. (c) Representative microphotographs of 3 *μ*m sections of thymus tissue stained with H&E (scale 200 *μ*m). The arrows indicate atrophy of the spinal region in diabetic animals induced with ALX or STZ. Data are presented as mean ± SEM, ^∗^*p* ≤ 0.05 (6-13 animals per group).

**Figure 5:**
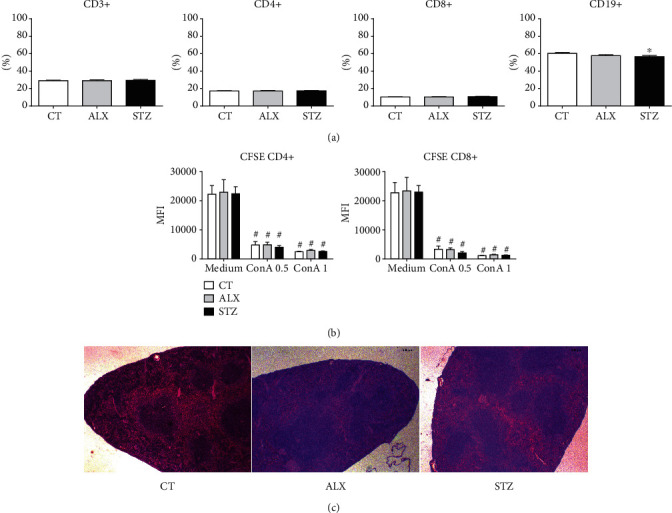
STZ has short-term diabetogenic impact on the population of B lymphocytes in the spleen. T1D was induced as described in Methods, and (a) spleens from the CT, ALX-, and STZ-treated groups were collected on the 15^th^ day, and the total spleen cells were characterized according to the cell surface markers CD3, CD4, CD8, and CD19 by flow cytometry. (b) Spleen cells from each group were also stained with CFSE, incubated with medium, Con A (0.5 and 1 *μ*g/mL—final concentration), for 72 hours, and had T cell proliferation evaluated by flow cytometry. (c) Representative microphotographs of 3 *μ*m sections of spleen tissue stained with H&E (scale 200 *μ*m). Data are presented as mean ± SEM, ^∗^*p* ≤ 0.05, and ^#^*p* < 0.05 versus respective control medium (6-13 animals per group).

**Figure 6:**
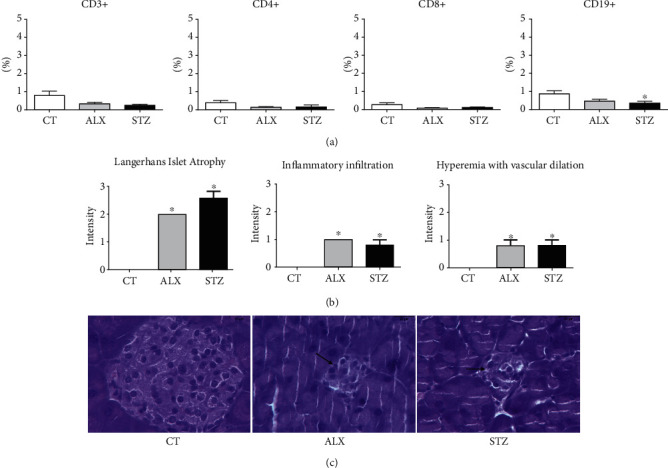
Short-term diabetogenic effects of ALX and STZ on the pancreas. T1D was induced as described in Methods, and (a) pancreases from the CT, ALX-, and STZ-treated groups were collected on the 15^th^ day, and the pancreatic cells were characterized according to the cell surface markers CD3, CD4, CD8, and CD19 by flow cytometry. (b) Evaluation of structural changes in the pancreas. (c) Representative microphotographs of 3 *μ*m sections of pancreas tissue stained with HE (scale 20 *μ*m). The arrows indicate atrophy of the islets of Langerhans in diabetic animals induced with ALX or STZ. Data are presented as mean ± SEM, ^∗^*p* ≤ 0.05 (6-9 animals per group).

Knowing that lymphocytes are the cell type most affected by the toxicity of ALX and STZ, T and B cell populations were determined in the thymus, spleen, and pancreas. It was found that the STZ-induced group showed a reduction in the percentage of total CD3^+^, CD4^−^CD8^+^ T lymphocytes, and CD4^+^CD8^+^ double-positive cells in the thymus (Figure 4(a)). A reduction in CD19^+^ B lymphocytes in the spleen (Figure 5(a)) and pancreas (Figure 6(a)) was also found. The ALX-induced group only showed changes in the thymus, including an increase in CD4^−^CD8^+^ cells, as well as CD19^+^ B lymphocytes (Figure 4(a)). This suggests that the diabetogenic agents influence lymphocyte subpopulations differently in the evaluated organs.

### 3.4. ALX and STZ Induced Morphological Changes in the Thymus and Pancreas, but Not in the Spleen

We also investigated whether ALX and STZ induced morphological changes in the pancreas, spleen, and thymus. In the pancreas, it was possible to identify, in both diabetic groups, atrophy of the islets of Langerhans (Figures 6(b) and 6(c)), inflammatory infiltration, and hyperemia with vascular dilation (Figure 6(b)). Being the pancreas a target organ of the diabetogenic agents, this may indicate that the destruction of the *β* cells was effective, followed by an inflammatory process as a side effect. The treatment seemed not to affect the integrity of the spleen in any diabetic group (Figure 5(c)). In the thymus, it was observed atrophy of the medullary region in both the ALX- and STZ-induced groups (Figures 4(b) and 4(c)). Enhanced death of lymphocytes/thymocytes, with an increase in Hassall's corpuscles and a decrease in the number of reticulum and epithelial cells in the ALX-induced group (Figure 4(b)), might indicate a change in the epithelial component of the thymus and the natural process of replacement of lymphocytes/thymocytes.

### 3.5. ALX and STZ Did Not Affect the *In Vitro* Proliferative Activity of CD4^+^ and CD8^+^ T Lymphocytes in the Spleen

In order to determine whether the diabetogenic agents were capable of influencing the functional status of lymphocytes, *in vitro* polyclonal proliferation was tested. Both CD4^+^ and CD8^+^ T lymphocytes showed no difference in activation induced by Con A after induction of T1D with ALX or STZ (Figure 5(b)), indicating that the agents did not interfere with *in vitro* cell proliferation.

### 3.6. STZ Caused Changes in the Immune Response Behavior *In Vivo* and *In Vitro* of Immunized Animals

**Figure 7:**
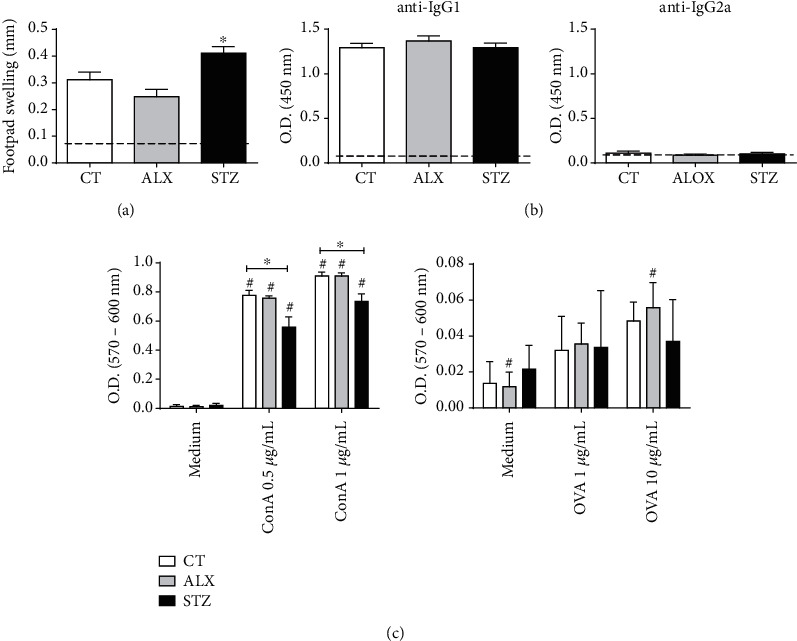
STZ short-term diabetogenic effects promote alterations in immune responses *in vitro* and *in vivo*. Mice were immunized with OVA, and 30 days later, T1D was induced as described in Methods. Fifteen days later, (a) antigen-specific paw edema was induced by footpad injection with OVA, (b) OVA-specific anti-IgG1 and anti-IgG2a were determined in the serum, and (c) *in vitro* spleen cell proliferation was performed by stimulation with medium, Con A (0.5 and 1 *μ*g/mL—final concentration) and OVA (1 and 10 *μ*g/mL—final concentration). Dashed line represents the mean of naïve mice not immunized with OVA. Data are presented as mean ± SEM, ^∗^*p* ≤ 0.05 (3-6 animals per group).

To assess whether ALX and STZ acted on the cellular and humoral acquired immune responses already established *in vivo*, mice were immunized with OVA and T1D was induced using both agents. Immunized mice from the STZ-induced group displayed an increased footpad thickness following challenge with OVA in comparison with mice from the CT and ALX-induced groups (Figure 7(a)). In addition, while all animals produced similarly increased levels of specific anti-OVA IgG1, anti-OVA IgG2a was virtually absent in immunized animals (Figure 7(b)). However, when the lymphocyte proliferative activity was evaluated *in vitro*, the immunized animals in the STZ-treated group showed a reduced polyclonal activation under Con A stimulation, but not OVA-specific activation, when compared to the CT and ALX-induced groups (Figure 7(c)). Together, these findings suggest an effect of STZ on the cell-mediated immune response of immunized animals while the humoral immune response did not seem to be affected.

## 4. Discussion

Animal models have been responsible for knowledge improvement and medical advances in several areas and from different perspectives. Chemically induced diabetes in rodent models has been developed to study not just the illness but also to better understand aspects of the condition. Because diabetes is a multifaceted and multifactorial disorder, animal models help to demystify different characteristics of its pathophysiology [13, 34, 35]. Since an immunopathogenic component in T1D had been previously described [36], much attention converged on choosing a model that could better contribute to the study of lymphocytes and their physiology. Although ALX and STZ are the most common pharmacological agents used to induce T1D, many toxicological effects on lymphoid organs and cells have been described [9, 10, 37–44].

Both agents were capable of maintaining the animals under T1D conditions during the whole study without causing reversible diabetes or inducing insulin or glucose tolerance effects [45, 46]. Therefore, it was possible to evaluate acute and chronic effects of T1D. Regarding blood cell counts, in the early phase of T1D, alterations in the proportion of immune cells were found in both the STZ- and ALX-treated groups. This initial phase was marked by an acute stress response which correlated to a reduction in the number of lymphocytes and monocytes and an increase in the percentage of granulocytes in animals treated with STZ during the first 30 days of diabetes induction, when compared to the CT group. The initial alteration in the blood cell count was spontaneously compensated during the study timeline and became very close to CT parameters, suggesting that there is a natural ability, even for diabetic animals, to adapt to this adverse condition. Interestingly, in the chronic phase of diabetes, the ALX group started to present subcutaneous tumor nodules after 90 days (data not shown), which might have contributed to the ~30% mortality observed in this group [31–33].

While some studies state that alterations in T1D animals are a consequence of drug pharmacodynamics [9, 10, 37–44], others claim that these changes are due to the hyperglycemic environment triggered by each agent [47–52]. Indeed, hyperglycemia affects the immune system [53] and can lead to defects in host immunity, such as impaired cell migration, phagocytosis, and intracellular killing [54]. A hyperglycemic state also increases the synthesis of advanced glycation end products (AGEs), secretion of proinflammatory cytokines, and oxidative stress pathways [55]. Whether these changes are connected to our findings remains to be determined.

Regarding the diabetogenic drugs used in this work, STZ might have some level of toxicity to bone marrow precursor cells since, in animals treated with this agent, a reduction in the percentage of lymphocytes and an increase in neutrophils were found. This toxic influence has already been described and may be explained by the STZ ability to cause endogenous suppression in the DNA of cells recently isolated from the bone marrow of healthy animals, showing that it might not be a change related to insulin deficiency [44]. This toxicity is reversible, as the harmful effects that ALX exerts on erythropoietic cells are compensated after one week of treatment [56]. In addition, a fluctuation in cell population might occur. Here, there was no difference in the number of neutrophils and lymphocytes in the bone marrow of the ALX-treated group when compared to the CT group, as confirmed by flow cytometer analyses conducted in bone marrow samples from ALX-treated BALB/C mice [57].

While changes in bone marrow precursors are thought to be correlated to STZ toxicity, alterations in red blood cell parameters in the ALX-treated group are most likely linked to an early exposure to a hyperglycemic environment and, later, to the renal function failure caused by the ALX ability to promote kidney injury [58, 59]. Moreover, since the ALX-treated group presented higher glycemia, this might be associated to a higher oxidative stress induction and vascular complications found in diabetic patients [60].

Lymphocytes from spleen and pancreas, however, were little affected, with only a slight reduction in the population of B lymphocytes in the STZ-treated group, which have already been described as sensitive to the agent [7]. *In vitro* activation of CD4^+^ and CD8^+^ T lymphocytes isolated from the spleen did not show proliferation impairment in any of the groups, suggesting low to no toxicity of the diabetogenic agents in secondary lymphoid organs. After immunization, however, *in vitro* activation of lymphocytes was shown to be more affected in animals induced with STZ. Similar results have been described by Itoh et al. [52], credited as an early deterioration in the immunological function [47], which later was shown to be a consequence of changes in the mitochondrial function that interfered with the metabolism of immune cells [61]. Lymphocyte proliferation impairment for animals treated with STZ was also described in other studies [9, 42, 43, 62], reinforcing the idea of STZ toxicity to lymphocytes.

Knowing that STZ might interfere with different immune cell populations and that lymphocytes present greater sensitivity to STZ, we showed a reduction in the total percentage of CD3^+^, CD8^+^, and double-positive CD4^+^CD8^+^ T lymphocytes in the thymus of the STZ group. These findings suggest that STZ impairs the development and maturation process of T lymphocytes. The double-positive stage precedes positive selection in the thymus that determines the differentiation either into CD4^+^ or CD8^+^ T cells through the continuous interaction between TCR and MHC/peptide complexes expressed by thymic epithelial cells [63]. Thus, it is possible that our findings are somehow related to the ability of the STZ to promote up or downregulation of MHC, as previously identified by Klinkhammer et al. who found that STZ induced an increase in class II antigen expression in different tissues, presumably due to its alkylating potential, which influenced MHC gene methylation [64]. Although we have not evaluated the expression of MHC in cells from the different organs, it is reasonable to expect that the aforementioned findings are also occurring in our experimental system. Nevertheless, the changes found in the ALX-treated group seem to make more sense in the context of diabetes, since a hyperglycemic environment leads to an increase in proinflammatory cytokines, causing deleterious effects in the body, followed by an increase in CD8^+^ T cells [65], which might be correlated with a profuse expression of MHC class I observed in autoimmune diabetes [66]. Corollary, there may be an enhancement in the number of B lymphocytes in order to restrain the damage, as they are responsible for inducing T lymphocyte tolerance in the thymus.

Atrophy of the thymus after treatment with diabetogenic agents was found in other studies with ALX [41, 67] and STZ [44, 68], which can partially explain our results. In addition, other thymic alterations were observed in the ALX-treated group, involving the lymphocyte/thymocyte natural replacement process by the epithelial component [24]. Here, in the ALX-induced group, however, these changes were not found when performing proliferation assays using mature lymphocytes.

Therefore, hyperglycemia itself may not be completely responsible for the changes found in the STZ-induced group, contrary to the findings by Sinzato et al. [51], indicating that a direct toxic effect of the agent on the lymphocyte population might occur, as stated in other studies [7, 8]. According to Muller et al., STZ is an analog of the glucose molecule and, therefore, may eventually be captured by glucose transporters in lymphocytes [7]. It is important to highlight that STZ is an agent that can possibly react with the genetic material of cells [69] and may also interfere with lymphocyte precursor populations in the bone marrow.

A chronic low-grade inflammation profile is a common feature of the T1D state [37, 65], contributing to the side effects of the disease. In our hands, however, this low-grade inflammation was not detected, as none of the agents was able to interfere with normal physiological concentrations of the cytokines evaluated in the spleen and pancreas, except for the decrease in the expression of IL-6 in the spleen and IL-1*β* in the pancreas, both in the group treated with STZ. Considering that IL-6 has the ability to promote activation and expansion of T cells, as well as differentiation of B cells [70], and that almost all stromal cells and cells of the immune system produce IL-6 [71], it is reasonable to assume that the reduction in their physiological concentration derives from the indirect effects of STZ, since the major producers of this cytokine do not belong to the lymphoid cell lineage (the cell population involved in the STZ toxicity). As such, changes in the feedback between lymphocytes and this cytokine may lead to impaired innate and adaptive immunity in viral, parasitic, and bacterial infections [72]. In spite of this, the decrease in IL-1*β* in the pancreas can be linked to intrinsic cellular defects of diabetic cells related to the induction of tolerance to stimulation [73] that somehow does not seem to be manifested in animals induced with ALX, although it might not be derived from a direct effect of STZ, since this agent has its toxicity mainly associated with lymphocytes.

We have observed a similar polyclonal spleen cell proliferation in the CT, ALX-treated, and STZ-treated groups after 15 days from T1D induction. Rubinstein et al. described similar findings at a similar time point and also at 1 and 3 months after T1D induction with STZ in rats, although a decreased proliferation was observed after 6 months [74]. On the other hand, Liu et al. observed that spleen cells of STZ-diabetic rats could proliferate more or less than cells from control rats depending on the Con A concentration used in the assay [75]. We have tested only two Con A concentrations in our assay with no differences observed and no later time points were evaluated. Regarding ALX-induced T1D, we have not found any study with a similar experimental approach to compare our findings.

In animals immunized with OVA, we observed an increased paw edema induced by antigenic challenge in the STZ group. Even though our findings contrast with those by Ishibashi et al., who found an increased antigen-specific paw edema in STZ-induced diabetic mice [76], there are many fundamental differences between their experimental protocol and ours: (I) Ishibashi et al. used sheep red blood cells (SRBCs) as an immunogen (a complex antigen with multiple epitopes), while we used OVA (a single protein with fewer epitopes); (II) immunization with SRBC was performed without adjuvant, while OVA immunization was done in the presence of squalene; (III) SRBC immunization was carried out after T1D induction with STZ, while OVA immunization was performed previous to STZ induction. Thus, a direct comparison between the two studies is not possible.

Regarding humoral responses in diabetic animals, the literature varies from impaired IgG responses following immunization [77, 78], to differences observed only after 6 months of T1D induction [74], to no differences [79]. Such findings differ from ours because we induced T1D after the immunization of the animals. Thus, once the antibody response is already established, the development of diabetes does not impact the production of IgG subclasses. Our results are in line with a previous case-control study that matched children with and without T1D, showing that no significant differences were observed in the antibody levels to pneumococcal serotypes, *Haemophilus influenzae*, as well as tetanus and diphtheria toxoids, that could be below the protective threshold between groups [80].

Choosing an appropriate animal model when planning a study is vital, as it is responsible for providing relevant and understandable scientific data. Although it is important to ensure the beneficial use of the model, there is not a perfect one yet and animals are not analogous to humans. Considering our study and the literature, animals induced with STZ would not be the best choice to study viral infections such as severe acute respiratory syndrome caused by coronavirus 2 (SARS-CoV-2). Here, where the model is only evaluated after T1D is established, it is not possible to conclude that the changes found were caused directly by the action of ALX or STZ or in response to T1D itself. Although it is clear that the immune response of animals induced with ALX or STZ is different after induction, further studies are needed to investigate the toxicity trigger of agents in immune cells during the development of T1D, to identify and separate direct effects of the agents and secondary effects of T1D. However, this does not compromise or diminish our findings, as it is necessary to consider that both groups of animals treated with ALX or STZ present a hyperglycemic environment in their conditions, which makes clear a greater influence of the STZ agent on aspects of the immune response.

## 5. Conclusions

In summary, we observed that both diabetogenic agents, ALX and STZ, influenced the architecture of lymphoid and nonlymphoid organs, as well as the relative proportion of certain cell populations. Nevertheless, the changes in the immune response profile *in vivo* and *in vitro* were more intense in the animals treated with STZ and in the early phase of diabetes. In the animals induced with ALX, there was a natural ability to balance most of the alterations observed. This was not observed in the animals induced with STZ, probably due to the greater toxicity of STZ to lymphoid organs, which may be associated with reactions in the STZ methyl group with the genetic material of lymphoid precursors.

## Figures and Tables

**Table 1:** STZ-treated animals showed an increase in the percentage of neutrophils and a decrease in lymphocytes in the bone marrow. Data are presented as percentage ± SEM, ^∗^*p* ≤ 0.05 (5-6 animals per group).

		Percentage ± SEM	
Cells (%)	CT	ALX	STZ
Blast	0.2 ± 0.2	0.0 ± 0.0	0.0 ± 0.0
Ring forms	3.3 ± 0.6	3.2 ± 1.0	1.5 ± 0.7
Segmented neutrophils	67.0 ± 1.9	75.6 ± 4.2	81.0 ± 2.1^∗^
Monocytes	2.0 ± 0.7	2.0 ± 0.5	1.0 ± 0.4
Lymphocytes	27.5 ± 2.3	19.2 ± 4.0	16.5 ± 2.5^∗^

## Data Availability

The data in this study are available upon reasonable request to the corresponding authors (Dr. Anderson Sá-Nunes, sanunes@usp.br or/and Joilson O. Martins, martinsj@usp.br).
